# Supplementary material for: Enhanced Antitumor Activity of Korean Black Soybean Cultivar ‘Soman’ by Targeting STAT-Mediated Aerobic Glycolysis
Source: Antioxidants (Basel). 2025 Feb 18;14(2):228. doi: 10.3390/antiox14020228 (PMC11852074; doi:10.3390/antiox14020228)
Supplement: Supplementary file 1 [file antioxidants-14-00228-s001.zip › antioxidants-3474066-supplementary.pdf]

Supplemental Table S1

Antibodies used in the immunoblotting (IB) and immunohistochemical (IHC) analyses:

Antibodies	Sources	Catalog no.	Dilution ratio
Anti-HK2	Santa Cruz	sc-130358	1:1,000 for IB
Anti-PKM2	Cell Signaling	3198	1:1,000 for IB
Anti-LDHA	Santa Cruz	sc-137243	1:1,000 for IB
Anti-Cyclin D1	Santa Cruz	sc-8396	1:500 for IB
Anti-c-Myc	Cell Signaling	18583	1:1,000 for IB
Anti-p-ERK1/2	Cell Signaling	9106	1:1,000 for IB
Anti-ERK1/2	Cell Signaling	9102	1:2,000 for IB
Anti-p-AKT	Cell Signaling	4060	1:1,000 for IB
Anti-AKT	Santa Cruz	sc-271149	1:2,000 for IB
Anti-p-p38	Santa Cruz	sc-166182	1:5,000 for IB
Anti-p38	Santa Cruz	sc-7972	1:1,000 for IB
Anti-p-c-Jun	Santa Cruz	sc-822	1:500 for IB
Anti-c-Jun	Santa Cruz	sc-74543	1:1,000 for IB
Anti-p-IκBα	Cell Signaling	2859	1:500 for IB
Anti-IκBα	Cell Signaling	4814	1:1,000 for IB
Anti-p-STAT1	Cell Signaling	7649	1:1,000 for IB,<br>1:200 for IHC
Anti-STAT1	Santa Cruz	sc-464	1:1,000 for IB
Anti-p-STAT3	Cell Signaling	9145	1:1,000 for IB
Anti-STAT3	Cell Signaling	4904	1:1,000 for IB
Anti-p-STAT5	Cell Signaling	4322	1:1,000 for IB
Anti-STAT5	Cell Signaling	94205	1:1,000 for IB
Anti-Ki-67	Abcam	ab16667	1:200 for IHC
Anti-NRF2	Proteintech	80593-I-RR	1:1000 for IB
Anti-SLC7A11	Cell Signaling	12691	1:1000 for IB
Anti-β-Tubulin	Sigma	T4026	1:5,000 for IB

Supplemental Table S2

Lists of primers for qRT-PCR analysis:

Gene	NCBI Accesion No.	Forward primer	Reverse primer	Amplicon size
ldha	XM_030242176.1	5'-TGTGGCAGACTTGGCTGAGA-3'	5'-CTGAGGAAGACATCCTCATTGATTC-3'	105
gapdh	NM_001411843.1	5'-CATCACTGCCACCCAGAAGACTG-3'	5'-ATGCCAGTGAGCTTCCCGTTCAG-3'	153

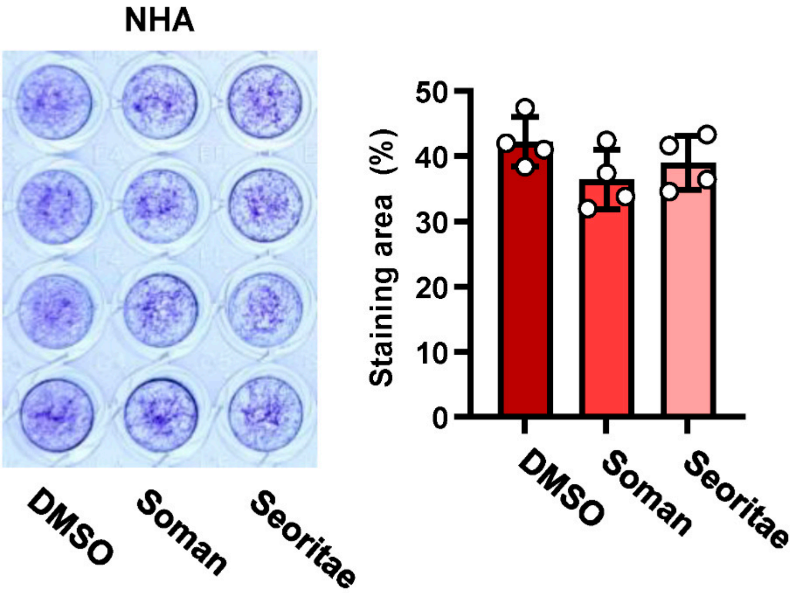

Supplemental Figure S1. Effects of Soman and Seoritae on the proliferation of NHA *in vitro*.

NHA cells were cultured with or without the indicated soybean extracts. The colonies were fixed with 10% formalin and stained with 0.1% crystal violet. Representative images (left panel) and graphs of the staining area (right panel) are shown.
